# Hematological parameters’ reference intervals in apparently healthy individuals in Saudi Arabia: a systematic review and meta-analysis

**DOI:** 10.3389/fmed.2025.1522492

**Published:** 2025-04-17

**Authors:** Naila Shaheen, Seena Thomas, Areej Almoghairi, Ahmed Alaskar

**Affiliations:** ^1^Division of Biostatistics, Department of Population Health, King Abdullah International Medical Research Center, Riyadh, Saudi Arabia; ^2^King Saud Bin Abdulaziz University for Health Sciences, Riyadh, Saudi Arabia; ^3^Ministry of National Guard Health Affairs (MNGHA), Riyadh, Saudi Arabia; ^4^Saudi Society of Blood and Marrow Transplant “SSBMT”, Riyadh, Saudi Arabia; ^5^Department of Pathology and Laboratory Medicine, King Abdul-Aziz Medical City, Riyadh, Saudi Arabia; ^6^King Abdullah International Medical Research Center, Riyadh, Saudi Arabia; ^7^Division of Adult Hematology and SCT, King Abdul-Aziz Medical City, Riyadh, Saudi Arabia

**Keywords:** hematological profile, hematological indices, reference intervals, reference values, reference range, healthy individuals, Saudi Arabia

## Abstract

**Background:**

The study aimed to perform a systematic review/meta-analysis of observational studies conducted in Saudi Arabia to identify the patterns of reported hematological parameters’ reference intervals (RIs).

**Methods:**

The literature search was performed using PubMed and Google Scholar. Observational studies that reported hematological parameters measured under normal physiological conditions in apparently healthy individuals were included. Studies conducted on non-healthy individuals and/or on pregnant women; those related to basic science, methodology, physiology, and non-physiological state; and those conducted on patients having co-morbidities were excluded. Studies on the pediatric population were also excluded from the meta-analysis. The methodological quality was assessed using standard critical appraisal instruments from the Joanna Briggs Institute (JBI) Critical Appraisal Checklist. R software was used to run the random-effects models. The results were reported as weighted mean differences and 95% confidence intervals. The complete blood count (CBC) parameter means were compared by sex using an independent samples *t*-test.

**Results:**

In total, 12 studies were included in the systematic review from all regions—Central (*n* = 5), Western (*n* = 5), Southern (*n* = 1), and Northern (*n* = 1). A total of eight (66%) studies focused on adults, and four (33.3%) studies reported a sample of adolescents/children. In addition, seven studies were not included in the meta-analysis for the following reasons: three studies reported only white blood cell (WBC) parameters, two studies had only abstracts available, and two studies involved newborns. High heterogeneity was demonstrated for all hematological parameters: red blood cell (RBC), hemoglobin (Hb), or mean corpuscular hemoglobin concentration (MCHC) (I^2^ = 100%); mean corpuscular hemoglobin (MCH) or hematocrit (HCT) (I^2^ = 99%); platelet (PLT) or mean corpuscular volume (MCV) (I^2^ = 98%); and WBC (I^2^ = 90%). The RBC (*p* = 0.009) and Hb (*p* = 0.0006) values were higher in the male participants. The PLT (*p* = <0.0001) values were higher in female participants. The remaining hematological parameters’ RIs were not statistically significant.

**Conclusion:**

The findings indicated some differences in the hematological parameters’ RIs reported across Saudi Arabia. We recommend establishing hematological parameters’ RIs based on the Saudi Arabian population to determine when to refer a patient with abnormal counts and to identify when to request further diagnostic work-up.

## Background

Hematological parameters are essential elements for assessing disease status (1). The complete blood count (CBC) is a routinely used test in clinics to establish disease diagnosis, the response to therapies, management, and follow-up (2). The currently used hematological parameters’ reference intervals (RIs) are based on the Western population (3). Normal ranges for hematological parameters’ RIs vary across populations. Hematological parameters’ RIs also vary by age, sex, ethnicity, environment, lifestyle, genetics, geographical location, analytical methods of testing, and diurnal variations (4). Hence, it is crucial to establish hematological parameters’ RIs based on each population. Hematological parameters’ RIs may also differ across regions of a country (5). Age-specific hematological parameters’ RIs have also been endorsed (6).

Extensive data have been reported for hematological parameters’ RIs in the Western population (3, 7). In the past decade, hematological parameters’ RIs have been reported in the Middle Eastern region (4, 5, 8–10). As reported in the literature, some hematological parameters, such as low neutrophil ranges, could be in the normal range for certain populations (11). Improper utilization of the CBC has been reported in the literature. It has also been reported that CBC results could be misinterpreted (12), which, in turn, impacts the quality of patient care. This finding highlights the need to establish hematological parameters’ RIs for each population.

The theory behind RIs was developed in 1969 by Grasbeck and Saris (13). The characterization and use of decision limits were published several years later by Galen and Gambino (14). However, despite the theory being well-defined, its clinical application continues to evolve; hence, the topic remains relevant in the hematology community. There are several reasons for this topic to be relevant: (i) a significant gap in the application of hematological parameters’ RIs exist, (ii) hematological parameters’ RIs need to be established based on individual populations, and (iii) some low values are not considered abnormal for certain populations (5, 11, 15–17), i.e., it is not necessary to consider values as abnormal if they are outside the normal range (13). The aim of this study was to perform a systematic review and meta-analysis of observational studies to identify the patterns of reported hematological parameters’ RIs in Saudi Arabia. The primary research question was “What are the ranges of the reported hematological parameters’ RIs among healthy adults in Saudi Arabia?”

## Methods

### Objectives

The objectives of the study were as follows: (i) to identify all studies published in Saudi Arabia reporting hematological parameters and (ii) to identify the reported hematological parameters’ RIs in healthy Saudi adults.

### Search strategy

The literature search was performed using PubMed and Google Scholar to identify articles published in English from 1960 to March 2023. The search strategy employed Medical Subject Headings and keywords that expressed the terms “hematological parameters,” “complete blood count,” “hematological profile,” “hematological reference intervals,” “reference intervals,” “reference ranges,” “reference values,” and “reference parameters,” crossed with the operator AND and terms such as “adults” and “Saudi Arabia.” The search strategy for PubMed is summarized in Table 1. Full-text articles were retrieved after reviewing the title and abstract.

**Table 1:** Search strategy in PubMed database.

Search key words	Results
“Reference Intervals” OR “Saudi Arabia”	129,161
“Hematological Profile”[Title/Abstract] OR “Reference Intervals”[Title/Abstract]	4,941
“Complete Blood Count” OR “Saudi Arabia”	645
“Hematological Profile” Middle East	352
*Filters: English	Total = 135,099

### Inclusion and exclusion criteria

#### Inclusion criteria

Observational studies conducted in Saudi Arabia, published in English until March 2023, and reporting hematological parameters were included in the systematic review. Studies that reported the hematological parameter measure outcome and the mean and standard deviation for hematocrit (HCT), red blood cells (RBCs), hemoglobin (Hb), mean corpuscular volume (MCV), mean corpuscular hemoglobin (MCH), mean corpuscular hemoglobin concentration.

(MCHC), white blood cells (WBCs), platelet (PLT), and WBC differentials (neutrophil, eosinophil, basophil, monocytes, and lymphocytes) were included in the meta-analysis.

#### Exclusion criteria

Any study conducted on non-healthy individuals and/or pregnant women; those related to basic science, methodology, physiology, and non-physiological states; those involving individuals with co-morbid conditions; and those conducted in other parts of the world and the Middle East region were excluded from the systematic review. Studies on the pediatric population were also excluded from the meta-analysis.

### Selection of studies

The authors (NS and AA) independently checked the titles and abstracts. The references were managed using Mendeley. The screening was performed to select articles published in English. The systematic review included primary research articles that reported a CBC test and included participants from all age groups. The meta-analysis was conducted for five studies only, with hematological parameters’ RIs data available for all CBC parameters in individuals aged ≥14 years (Table 2).

**Table 2:** Studies flow chart.

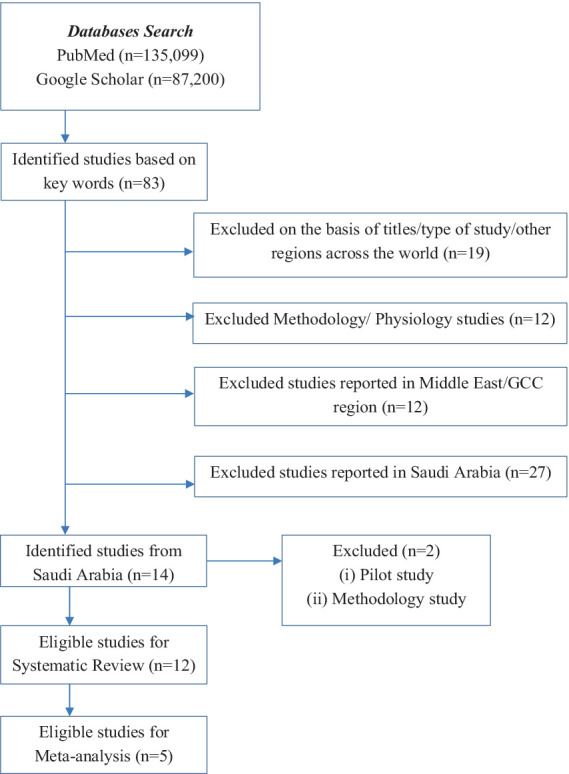

### Assessment of methodological quality

The selected studies were assessed for methodological quality by two independent reviewers (NS and AA) using standard critical appraisal instruments from the Joanna Briggs Institute (JBI) Critical Appraisal Checklist (18).

**Figure 1:**
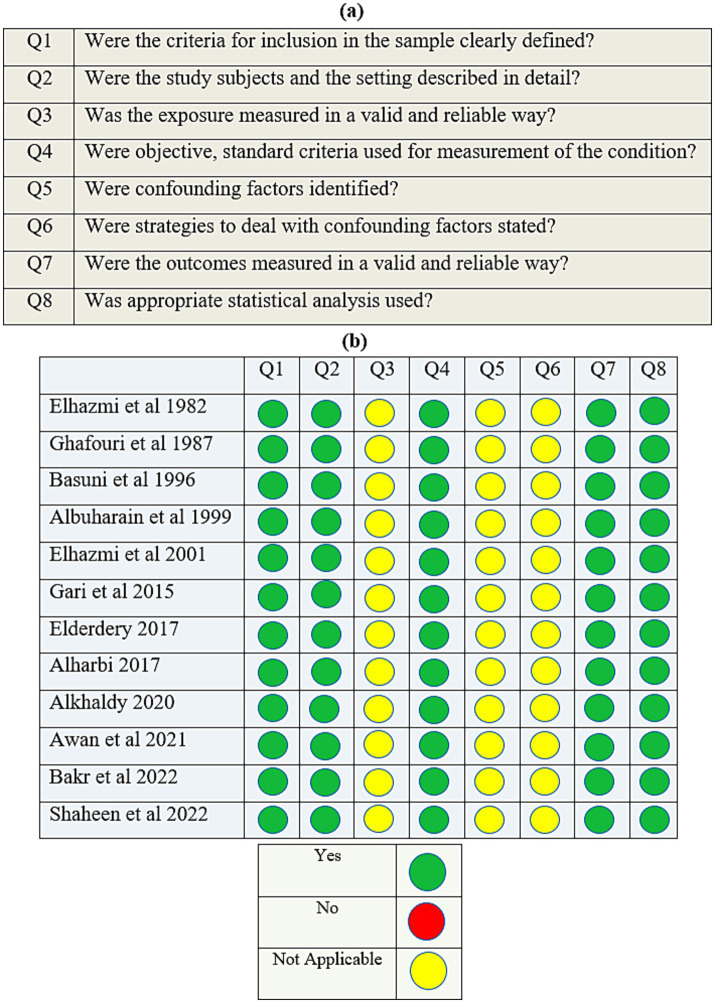
**(a)** The Joanna Briggs Institute’s (JBI) critical appraisal checklist for analytical cross-sectional studies; **(b)** quality assessment of the cross-sectional studies.

The methodological assessment criteria were based on the eight elements of the JBI Critical Appraisal Checklist (Figures 1a,b). A cut-off score of at least 60% was considered the inclusion criterion. The study complied with the Meta-analyses Of Observational Studies in Epidemiology (MOOSE) Checklist (19).

### Data extraction

Data were extracted by the authors using a standardized data extraction sheet. The extracted data included the year of publication, the location of the study, sample size, hematological parameters’ RIs, and mean and standard deviation both for male and female participants (Table 3).

**Table 3:** Characteristics of the included studies in the systematic review and meta-analysis.

Serial number	Author	Publication year	City; region	Study design	Study subjects	Number of subjects	M/F	Age range	CBC analyzer	Included in meta-analysis	Ref
1	El-Hazmi et al	1982	Riyadh; Central region	Cross-sectional	Young adults	804	578/226	20–29	Not available	No (abstract only)	(23)
2	Ghafouri et al	1987	Jeddah; Western region	Cross-sectional	Birth/Adolescents	1,673	843/830	-	Not available	No (abstract only)	(27)
3	Bassuni et al	1996	Abha; Southern region	Cross-sectional	Newborns	578	-	-	Electronic Coulter counter (Model S + IV)	No (Newborns)	(29)
4	Buhairan et al	1999	Riyadh; Central region	Cross-sectional	Adults	300	150/150	20–40	Coulter Counter STK-S	Yes *N* = 300	(36)
5	El-Hazmi et al	2001	Riyadh; Central region	Cross-sectional	Children	1,526	-	1–15	Coulter Counter ZF6	Yes *N* = 97 (Results for only group with age >14–15 were included)	(25)
6	Gari et al	2015	Jeddah; Western region	Cross-sectional	Adults	100, Saudi *n* = 69	-	18–55	BD FACS Canto II flow cytometer (San Jose, California, USA)	No (only reported overall WBC parameters)	(17)
7	Elderdery et al	2017	Al-Jouf; Northern region	Cross-sectional	Adults	2,040	1,152/888	17–28	BS-320	Yes *N* = 2040	(30)
8	Alharbi et al.	2017	Jeddah; Western region	Cross-sectional	Newborns	2,163	-	-	BS-320	No (Newborns)	(28)
9	Alkhaldy et al	2020	Jeddah; Western region	Cross-sectional	Adolescents/Adults	21,550	-	12–60	Sysmex automated analyzers (Sysmex corporation, Kobe, Japan)	No (only reported WBC parameters)	(15)
10	Awan et al	2021	Jeddah; Western region	Cross-sectional	Adolescents/Adults	91,880	-	13–60	Sysmex automated analyzers (Sysmex Corporation, Kobe, Japan)	No (only reported WBC parameters)	(11)
11	Bakr et al	2022	Riyadh; Central region	Cross-sectional	Adults	637	-	15–65	SYSMEX XN-10 instrument (Sysmex Corporation, Kobe, Japan)	Yes *N* = 637	(26)
12	Shaheen et al	2022	Riyadh; Central region	Cross-sectional	Adults	1,388	-	18–55	ADVIA2120i (Siemens Healthcare Diagnostics, Deerfield, IL, USA) and Cell-Dyn Sapphire (Abbott Laboratories, IL, USA),	Yes *N* = 1,388	(5)

### Statistical analysis

For continuous variables, weighted mean differences (SD) and 95% confidence intervals were estimated. Depending on the degree of study heterogeneity, a random-effects model was employed to obtain the pooled estimate of the results. The I^2^ statistic and corresponding *p*-value were used to evaluate the degree to which statistical heterogeneity in the meta-analyses was caused by variations between the studies rather than by chance. Forest plots were used to represent the type of statistical heterogeneity. The data for CBC parameters were normally distributed, except for neutrophils. The mean values of the parameters were compared between male and female individuals using an independent samples *t*-test. All statistical analyses were performed using R software version 4.3.2.

### Publication bias

Funnel plots are graphical tools commonly used in observational studies to assess publication bias and evaluate the presence of small study effects. These plots provide a visual representation of the relationship between effect size estimates from individual studies and their corresponding precision measures, such as standard errors or sample sizes (20). In a funnel plot, each point on the graph represents a study, with the effect size displayed on the horizontal axis and the precision measure on the vertical axis. In the current study, the funnel plot displays mean values on the horizontal axis and standard errors on the vertical axis. A vertical line through the center of the plot typically represents the true effect size. The shape of the funnel is determined by the inherent variability in effect sizes due to sampling error, with larger studies having smaller standard errors and thus being more precise (21). In the absence of publication bias, the points on the plot are expected to be symmetrically distributed around the true effect line. Studies in the upper portion of the plot with low standard errors are expected to cluster closer to the true effect line, resulting in a narrower funnel. In the lower portion of the figure, the funnel opens up, and with increasing standard errors, effect sizes are expected to scatter more widely to the left and right of the true effect. Supplementary Figures S1, S2 represent the funnel plots. Egger’s test was performed to quantify publication bias. Supplementary Table S1 shows the absence of publication bias, as the *p*-values were > 0.05 for all of the reported variables (21).

## Results

### Selected studies

A total of 1,35,099 articles were identified after searching the PubMed database (Table 1). The details of exclusions are provided in Table 2. In total, 13 studies have reported hematological parameters’ RIs in Saudi Arabia from 1982 to present. A latest published study focused on methodology (22) and a pilot study published in 1982 (19) were excluded. A total of 12 studies reporting hematological parameters’ RIs under normal physiological conditions across all age groups were included in the systematic review. The published studies originated from all regions: Central (*n* = 5), Western (*n* = 5), Southern (*n* = 1), and Northern (*n* = 1). The majority of the studies, eight (66%), focused on adults, while four (33.3%) studies reported a sample of adolescents or children (Table 3).

### Characteristics of studies

The characteristics of the included studies are summarized in Table 3. A total of five studies were reported from Riyadh (Central region) (5, 23–26), five from Jeddah (Western region) (11, 15, 17, 27, 28), one from Aljouf (Northern region) (29), and one from Abha (Southern region) (30). All studies were cross-sectional and included both male and female individuals. The twelve studies were included in the systematic review. A total of seven studies were not included in the meta-analysis for the following reasons: three studies reported only WBC parameters, two studies had only abstracts available, and two studies involved newborns (Table 3).

### Meta-analysis outcome

**Figure 2:**
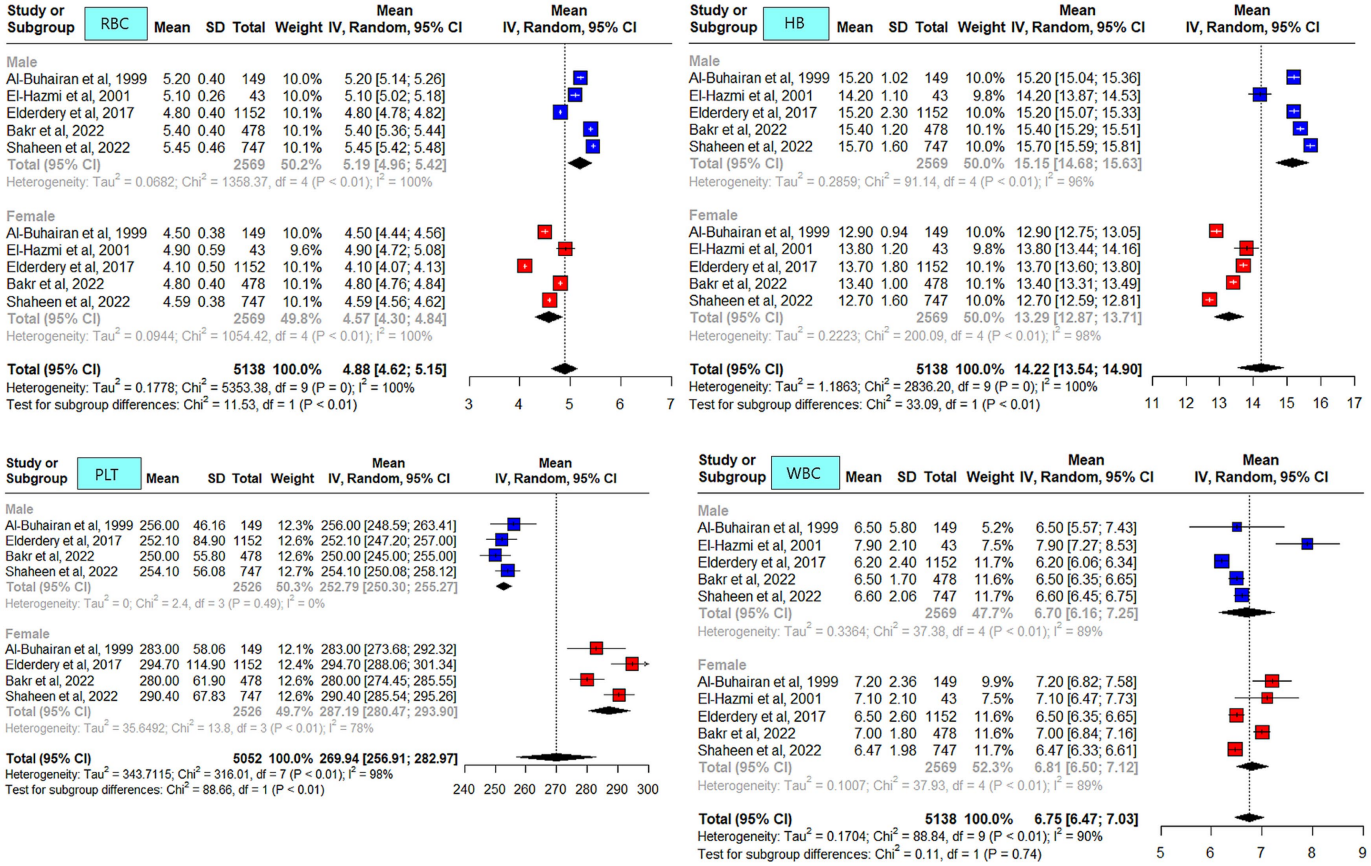
Mean red blood cell (RBC), hemoglobin, platelets (PLT), and white blood cell (WBC) counts in apparently healthy male and female individuals in Saudi Arabia.

**Figure 3:**
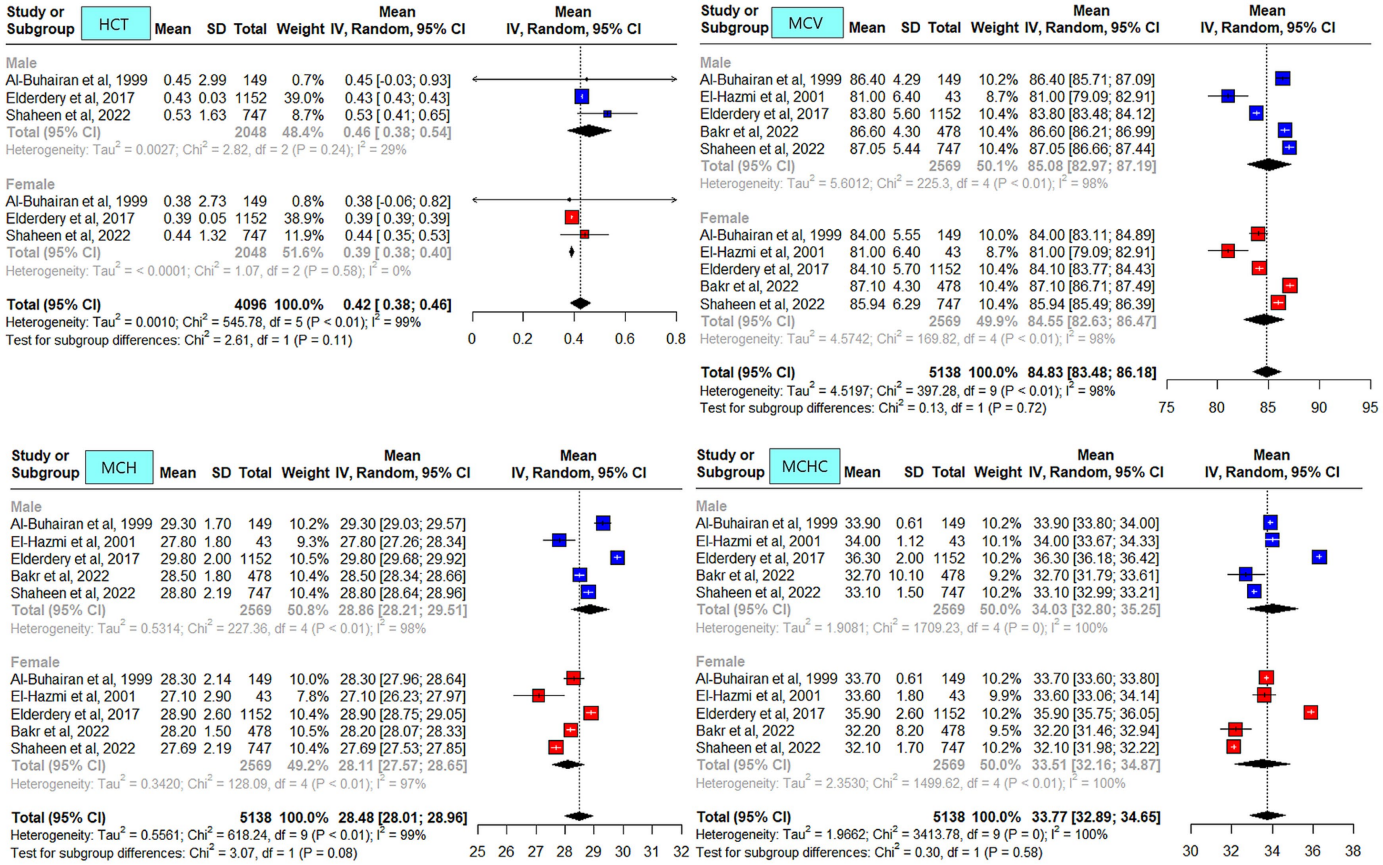
Mean hematocrit (HCT), mean corpuscular volume (MCV), mean corpuscular hemoglobin (MCH), and mean corpuscular hemoglobin concentration (MCHC) in apparently healthy male and female individuals in Saudi Arabia.

**Figure 4:**
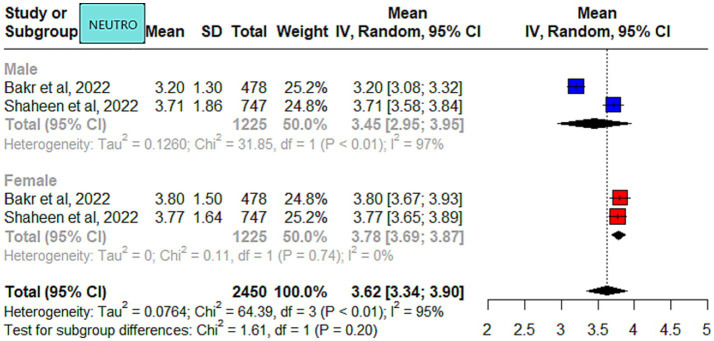
Mean neutrophils in apparently healthy male and female individuals in Saudi Arabia.

The meta-analysis results of the data driven from the five included studies are summarized in Figures 2–4, presenting data for RBC, Hb, PLT, WBC, HCT, MCV, MCH, and MCHC. High heterogeneity was observed across all hematological parameters: RBC, Hb, or MCHC (I^2^ = 100%); MCH or HCT (I^2^ = 99%); PLT or MCV (I^2^ = 98%); and WBC (I^2^ = 90%). The RBC (*p* = 0.009) and Hb (*p* = 0.0006) values were higher in male individuals. The PLT values (*p* = <0.0001) were higher in female individuals. The remaining hematological parameters showed no statistically significant sex differences. The pooled mean estimates for all hematological parameters are summarized in Table 4.

**Table 4:** The estimated pooled mean and 95% CI of hematological parameters’ reference intervals among apparently healthy people in Saudi Arabia.

Complete blood count parameters	Male		Female	
	Mean	95%CI	Mean	95%CI
RBCs (10^12^/l)	5.19	4.96–5.42	4.57	4.30–4.84
Hemoglobin (g/dl)	15.15	14.68–15.63	13.29	12.87–13.71
Platelets (10^6^/mm^3^)	252.79	250.3–255.2	287.18	280.4–293.9
WBCs (10^9^/l)	6.70	6.16–7.25	6.81	6.50–7.12
Hematocrit (%)	0.46	0.38–0.54	0.39	0.38–0.40
MCV (fl)	85.08	82.97–87.19	84.55	82.63–86.47
MCH (pg)	28.86	28.21–29.51	28.11	28.21–29.51
MCHC (g/dl)	34.03	32.80–35.25	33.51	32.16–34.87

### Risk of bias and sensitivity analysis

Sensitivity analysis was not carried out as it was not applicable due to (i) the included studies having no differences in terms of quality and (ii) the absence of specific sub-groups.

## Discussion

The importance of hematological parameters’ RIs can be ascertained by the fact that they are crucial not only for disease diagnosis but also for disease monitoring. It is important to consider that hematological parameters’ RIs can be affected by sex, age, ethnicity, altitude, and lifestyle (31). The literature extensively reports on hematological parameters’ RIs in the Western population (3, 32). In the past decade, a few studies have been reported in the Middle Eastern region (4, 8–10). Studies have also been reported across Saudi Arabia describing hematological parameters’ RIs. To the best of our knowledge, this is the first systematic review and meta-analysis reporting hematological parameters’ RIs in Saudi Arabia.

When locally published studies were compared, sex differences were reported in all studies. Overall, the reported hematological parameters’ RIs (RBC, Hb, HCT, MCV, and MCH) were higher in male individuals compared to female individuals. The WBC and platelet counts were higher in female individuals compared to male individuals. These observations are consistent with those reported in the literature (10, 32–35).

In the current meta-analysis, the average Hb level in male and female individuals was similar across studies (5, 25, 26, 30, 36). The Hb level reported by El-Hazmi et al. was one unit lower, which could most likely be attributed to participants’ age range of 14–15 years (25). The Hb RI was reported to be lower in Saudi female individuals compared to other Middle Eastern populations and Caucasians (5). One possible explanation for the reported variation could be the menstrual cycle duration across populations (37). Another possible explanation is dietary habits, including nutritional factors (e.g., iron-enriched food), which can lead to iron deficiency anemia. A study reported lower average food intake compared to the dietary requirements in Saudi women (38). A study conducted on Saudi female students reported that 64% were anemic despite being on iron supplements (39). Another study from the Western region reported an overall prevalence of anemia of 39%, with higher rates in female individuals 68%. However, it could not be ascertained whether the participants were only locals (40). The lower limit of the pooled mean Hb in male individuals exceeded values reported for the Western population (1, 32, 41).

PLT was consistently higher in female individuals across the studies compared to male individuals, and this finding might be related to hormonal changes due to estrogen-promoting platelet production (42), the menstrual cycle (43), or genetic variability (44). Low iron stores in the body can also be a contributing factor (45).

A study from Riyadh, in the Central region, reported slightly higher WBC counts in male individuals compared to other studies (25). This difference could be attributed to the younger age of the participants (14–15 years) compared to the other four studies. It is most likely related to the development of the immune system because the study population was from the pediatric age group. Of the included studies in the meta-analysis, only two were qualified to report neutrophils. Slight differences were observed in the neutrophil counts for male individuals reported by the two studies (5, 26).

Benign ethnic neutropenia (BEN) was first reported in 1941 by Forbes et al. and is common in African, African Caribbean, Ethiopian, Yemenite Jew, and Arab populations (16). BEN is commonly reported in the Arab population (17, 46). It is a commonly observed finding, based on our clinical practice observations and received referrals from other disciplines. The reported BEN prevalence ranged from 11 to 23% across Saudi Arabia (5, 11, 17). However, the results cannot be generalized, either due to low sample size (11) or the use of different cut-offs based on hospital reference intervals in the studies. Moreover, in the current meta-analysis, only two studies reported neutrophil counts, so it is not justified to comment further. Caution should be exercised when requesting additional diagnostic work-up as this may subject patients to unnecessary testing and anxiety.

Sex-related differences were not reported in newborns or children. As part of normal physiological changes over time, variations in hematological parameters’ RIs were reported in a study conducted on individuals from birth through adolescence (27). Hematological parameters’ RIs were also reported to differ between low and high altitudes.

It has been previously reported that hematological parameters’ RIs could vary across regions in Saudi Arabia (5). The differences could be attributed to several factors (11). However, in the current meta-analysis, it is difficult to comment on the differences in hematological parameters’ RIs across regions, as the majority of the studies (4/5) were reported from the Central region.

Smoking is another factor that has been reported to impact the levels of WBC, HB, MCV, and MCHC, typically increasing them (47). In the current meta-analysis, smoking was either not reported in the included studies (25, 26, 36) or was excluded (30). The studies showed an increase in RBC parameters in male individuals over time in the Saudi population (5, 26, 30, 36), which could be attributed to the increase in smoking prevalence in Saudi Arabia over the years (48). Notably, the prevalence of smoking (14%) in Saudi Arabia was reported to be higher in male individuals (25%) compared to female individuals (1.9%) (49).

### Challenges in study comparison

The comparison across the studies was challenging due to variations in study settings, sample sizes, analytic conditions—including the technology used for blood counters—assessed parameters, the populations under study, the methods for selecting the study population, age ranges, and hematological parameters’ RIs, which were based on local laboratory standards for each study. Although the studies used terms such as “volunteers,” “healthy individuals,” and “participants coming for routine check-ups,” it was still challenging to identify a healthy individual in the Saudi population due to the high prevalence of disorders related to Hb, iron deficiency anemia, and endemic viral infections.

### Recommendations

Several countries have established RIs based on their populations (41, 50). It is well documented in the literature that reported RIs vary across populations (51, 52). Therefore, we recommend establishing hematological parameters’ RIs based on the Saudi population on a broader scale. Special precautions should be taken when designing studies reporting hematological parameters’ RIs, taking into consideration factors such as geographical region, altitude, genetic factors, lifestyle, risk factors, and the baseline health status of participants. Future studies focusing on hematological parameters should also consider confounders such as smoking status and iron store assessments.

The CBC is a commonly requested laboratory test, but it can be misinterpreted. Caution should be exercised when interpreting CBC results as hematological parameters’ RIs vary across laboratories. It is important to recognize that inappropriate interpretation of hematological parameters’ RIs might lead to unnecessary further investigations or a failure to identify the underlying disease. This finding, in turn, can affect the quality of patient care and have an impact on the economy.

## Conclusion

The findings of the current review indicated some differences in the reported hematological parameters’ RIs across Saudi Arabia. We recommend establishing hematological parameters’ RIs based on the Saudi population. We also emphasize the need for a consensus (i) to establish the cut-off value for hematological parameters’ RIs for the Saudi population, (ii) to determine when to refer a patient with abnormal counts, and (iii) to identify when to request further diagnostic work-up.

## Data Availability

The raw data supporting the conclusions of this article will be made available by the authors, without undue reservation.

## Supplementary material

SUPPLEMENTARY FIGURE S1 Publication bias for CBC parameters [Mean Red Blood Cells (RBC), Hemoglobin, platelets (PLT), and White Blood Cell Count (WBC)].

SUPPLEMENTARY FIGURE S2 Publication bias for CBC parameters [Mean Hematocrit (HCT), Mean Corpuscular Volume (MCV), Mean Corpuscular Hemoglobin (MCH), Mean Corpuscular Hemoglobin Concentration (MCHC)].

SUPPLEMENTARY Table 1 Publication Bias across studies.
