# Supplementary figures and images for: Hematological parameters’ reference intervals in apparently healthy individuals in Saudi Arabia: a systematic review and meta-analysis
Source: Front Med (Lausanne). 2025 Apr 17;12:1522492. doi: 10.3389/fmed.2025.1522492 (PMC12043445; doi:10.3389/fmed.2025.1522492)

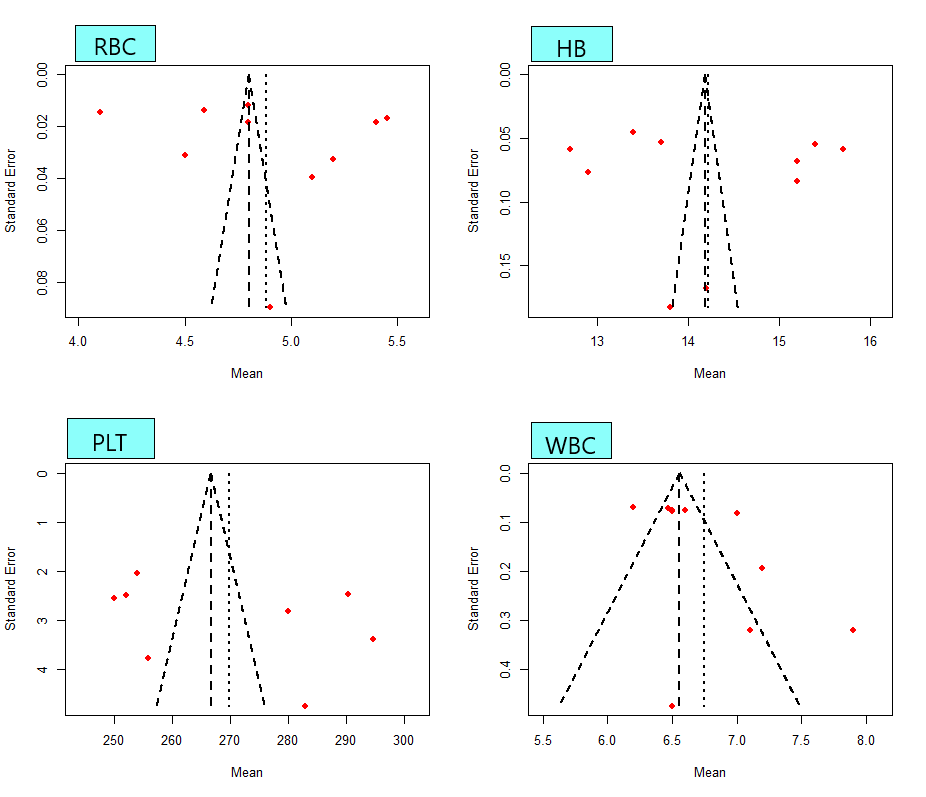

Supplement: SUPPLEMENTARY FIGURE S1 — Publication bias for CBC parameters [Mean Red Blood Cells (RBC), Hemoglobin, platelets (PLT), and White Blood Cell Count (WBC)]. [file Image_1.tiff]

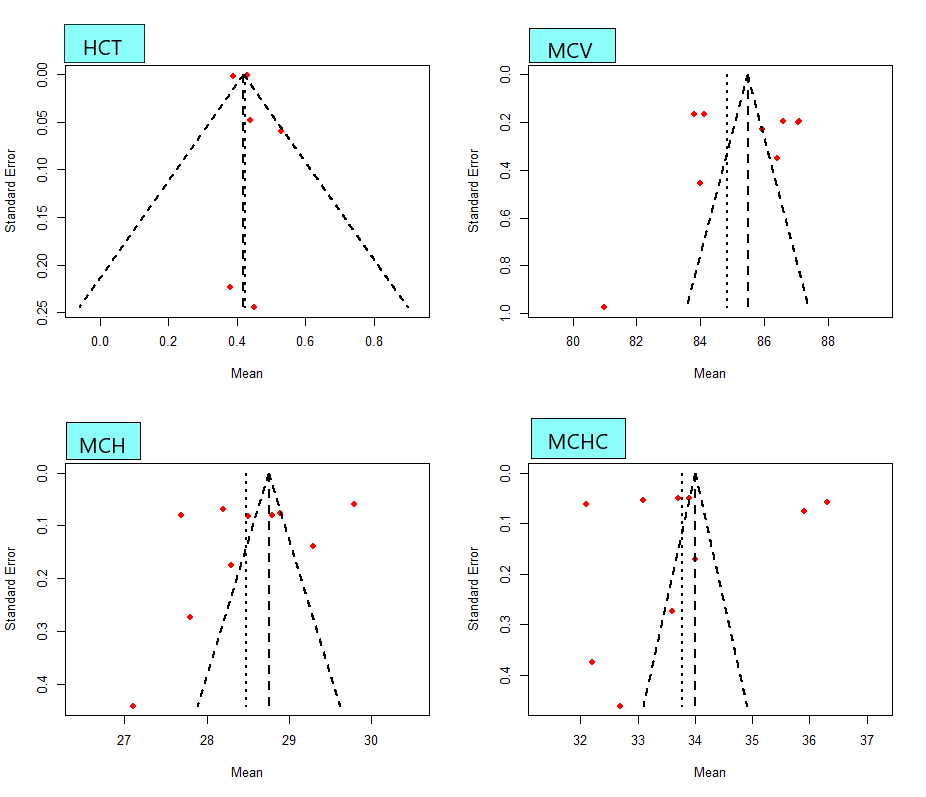

Supplement: SUPPLEMENTARY FIGURE S2 — Publication bias for CBC parameters [Mean Hematocrit (HCT), Mean Corpuscular Volume (MCV), Mean Corpuscular Hemoglobin (MCH), Mean Corpuscular Hemoglobin Concentration (MCHC)]. [file Image_2.tiff]
