# Supplementary material for: Hematological parameters’ reference intervals in apparently healthy individuals in Saudi Arabia: a systematic review and meta-analysis
Source: Front Med (Lausanne). 2025 Apr 17;12:1522492. doi: 10.3389/fmed.2025.1522492 (PMC12043445; doi:10.3389/fmed.2025.1522492)
Supplement: SUPPLEMENTARY Table 1 — Publication Bias across studies. [file Table_1.docx]

**Supplemental Table 1: Publication Bias across studies**

| **Parameter** | **Test statistic value** | **p-value** |
| --- | --- | --- |
| HB | 0.61 | 0.542 |
| HCT | -0.08 | 0.936 |
| MCH | -0.19 | 0.842 |
| MCHC | 0.14 | 0.887 |
| MCV | -0.96 | 0.339 |
| PLT | 0.32 | 0.747 |
| RBC | -0.69 | 0.487 |
| WBC | -0.04 | 0.964 |
